# The effect of early broad-spectrum versus delayed narrow-spectrum antibiotic therapy on the primary cure rate of acute infection after osteosynthesis

**DOI:** 10.1007/s00068-019-01182-6

**Published:** 2019-07-16

**Authors:** Pien Hellebrekers, Michiel H. J. Verhofstad, Luke P. H. Leenen, Hilal Varol, Esther M. M. van Lieshout, Falco Hietbrink

**Affiliations:** 1 Department of Surgery, University Medical Center Utrecht, P.O. Box 85500, Heidelberglaan 100, 3508 GA Utrecht, The Netherlands; 2 Trauma Research Unit, Department of Surgery, Erasmus MC, University Medical Center Rotterdam, Rotterdam, The Netherlands

**Keywords:** FRI, Treatment, Antibiotics, ORIF, Infection, Fracture

## Abstract

**Purpose:**

Infection near metal implants is a problem that presents challenging treatment dilemmas for physicians. The aim of this study was to analyse the efficacy of two treatment protocols for acute fracture-related infections.

**Methods:**

Seventy-one patients in two level-1 trauma centres in the Netherlands were retrospectively included in this study. These trauma centres had different standardised protocols for acute infection after osteosynthesis: 39 patients were selected from protocol *A* and 32 from protocol *B*. Both protocols involve immediate surgical debridement and soft tissue coverage, but differ in antibiotic approach: (A) immediate empirical combination antibiotic therapy with rifampicin, or (B) postponed (1–5 days) targeted antibiotic therapy. The primary outcome of these protocols was success, defined as a fracture healing in the absence of infection. The secondary outcome was antibiotic resistance patterns. Logistic regression was conducted on patients and treatment-related factors in association with primary success.

**Results:**

Primary success was achieved in 72% of protocol *A* patients, in 47% of those in protocol *B* (*P *= 0.033), and with prolongation of treatment success was achieved in 90% and 78% of patients, respectively. Protocol *A* exhibited a better primary success rate (adjusted OR 3.45, CI 1.13–10.52) when adjusted for age and soft tissue injury. There was no significant difference in antibiotic resistance between the two protocols.

**Conclusion:**

Both protocols yielded high overall success rates. Immediate empirical antibiotics can be used safely without additional bacterial resistance and may contribute to increased success rates.

## Introduction

Fracture-related infection (FRI) is a serious problem after operative fracture management. It is accompanied by increased morbidity, prolonged hospital stays, an increased number of operations and thereby a significant increase in healthcare costs [1]. FRI has an incidence of approximately 1–2% in closed fractures and increases to up to 50% in severe open fractures [2–4].

Treatment regimens for prosthetic joint infections (PJI) are becoming increasingly well defined, but PJI differs significantly from FRI in terms of the soft tissue problems and the need for fracture stability that accompany it, and evidence-based guidelines for FRI are currently insufficient [5]. Unfortunately, infection near orthopaedic implants seems to be highly complex and poorly described. This has resulted in a diverse collection of treatment protocols, with recommendations only for specific circumstances. Although aggressive surgical debridement seems to be embedded in most approaches, a consensus on standardised antibiotics or the retention of orthopaedic implants has yet to be reached [3, 6–8].

In a previous study, the effect of an aggressive standardised approach to FRI, which consisted of immediate surgical debridement with hardware retention and combination antibiotics with rifampicin, produced promising results. High overall success rates were achieved, but the implications of empirical antibiotics in the context of emerging resistance remain the subject of debate [9].

To contribute to the knowledge gap on this subject, we compared two standardised regimens that shared the philosophy of a uniform approach to all acute FRI, including all anatomic sites and fixation types. Both consisted of immediate aggressive debridement for source control, retention of orthopaedic implants and early soft tissue coverage, but differed in their antibiotic treatment guidelines. The aim was to analyse the effect of the regimens in terms of success rates, microbiological aspects and emerging resistance.

## Methods

### Patient identification

A retrospective cohort study of patients who had been treated for FRI was performed in two level-1 trauma centres in the Netherlands. The inclusion period was from January 1, 2008 (hospital B) or January 1, 2011 (hospital A) until December 31, 2014. Patients were identified by diagnosis codes, operation registers and antibiotic administration. Patients of between 17 and 75 years of age with acute symptoms (< 3 weeks) of early and delayed FRI (< 10 weeks after fracture fixation) who had been treated according to the standardised treatment protocols were included in the study [10]. Infection was diagnosed through a combination of clinical and laboratory findings (redness, swelling, wound discharge, elevated leukocytes and/or CRP), purulent drainage, peroperative pus or microbiological identification. Exclusion criteria were as follows: symptoms for > 3 weeks upon presentation (due to biofilm maturation), chronic infections and/or immune suppressive state (e.g., chronic immunosuppressive medication, immune deficiencies) and removal of orthopaedic implants at first debridement. Patient and treatment characteristics were collected from patients’ files. This study was reviewed, and a waiver was provided by the local Medical Ethics Review Committee (METC), no. 14/343. All procedures were performed in accordance with the principles of the Declaration of Helsinki.

### Treatment protocols

Both protocols were followed in each hospital independently of the type of infection ([sub]acute or early/delayed), location or fixation type.

### Surgical treatment

In all patients, an early and thorough surgical debridement was performed as soon as possible after a (suspected) diagnosis of FRI. Surgical debridement was repeated as often as required by the attending surgeon. Orthopaedic implants were retained when they were stable and sufficient. Primary closure after debridement was the aim in every patient. In protocol *B*, a wound drain was used at the surgeon’s preference, while in protocol *A*, no drains were used. When primary closure could not be achieved, the alternative treatment in protocol *A* was to apply a vacuum dressing with secondary wound closure. In larger defects, secondary flap or skin transplants were considered. In protocol *B*, direct flap transplantation with or without a vacuum dressing was the preferred treatment.

### Antibiotic treatment

*Protocol A* Empirical antibiotic therapy started immediately after the first debridement when cultures had been obtained. This consisted of 10 days of intravenous administration of antibiotics. The antibiotic therapy began with a combination of vancomycin and rifampicin. Vancomycin was chosen because of its activity against most common pathogens of FRI and its synergetic effect with rifampicin [11]. Vancomycin was adjusted when culture and susceptibility data were available. Rifampicin was continued if not otherwise contra-indicated. After the intravenous administration period, a combination of antibiotic therapy including rifampicin was orally continued for ten additional weeks, depending on the culture and susceptibility data.

*Protocol B* Antibiotic therapy started after culture data had become available (1–5 days) and consisted of a 14-day targeted intravenous administration period, followed by 4 weeks of oral antibiotic treatment. The type and administration (intravenous or oral) of antibiotics depended on bacterial identification and susceptibility data. Rifampicin was only administered when all cultured bacteria were susceptible.

In both protocols, patients were given a peroperative antibiotic prophylaxis, which in most cases consisted of a one-time administration of cephalosporin after cultures had been sampled. In the case of repeated debridement, antibiotic therapy was continued in both protocols according to the culture results from the first debridement and was adjusted only when necessary. No additional preoperative antibiotic prophylaxis was administered at redebridement.

### Microbiology

From all patients, cultures (tissue, swab and fluid) were obtained during initial surgical debridement and were repeated with each additional surgery. Isolates were cultured and identified using standard techniques. Susceptibility testing was performed on isolates using the Phoenix automated susceptibility testing system, disk diffusion and/or E-test strips.

Initial resistance was defined as resistance before administration of the applicable antibiotic, while intrinsic resistance was not considered to be initial resistance. Follow-up resistance was defined as resistance after the administration of the respective antibiotic, regardless of whether that pathogen had been cultured before. Overall resistance was defined as the combination of both abovementioned types.

### Outcome measures

The primary outcome measure was success. Primary success was defined as resolved signs and symptoms of infection and fracture consolidation on radiological follow-up, as assessed by the treating physician after a single protocol run. In some cases, fracture consolidation as assessed on routine X-rays took longer than the duration of one protocol run. Therefore, the moment of fracture consolidation on follow-up X-rays was counted as the moment of success. Inflammatory markers were not part of the criteria for success because of their low sensitivity and specificity in the diagnosis of FRI, and they are not routinely measured in either protocol [12]. Overall success was defined as success after the prolongation of antibiotic treatment or repetition of the protocol (including possible redebridement and/or antibiotics). A protocol was considered to be a failure if orthopaedic implants were removed, if a case of non-union occurred, or if amputation was performed.

The secondary outcome measure was the emergence of resistance against administered antibiotics. Microbiological characteristics and susceptibility patterns were described.

### Statistical analysis

Analyses were conducted using SPSS 24 (IBM, New York, NY). Baseline characteristics were compared between protocols, and descriptive analysis was used to report data per group. The statistical significance of continuous data—which were all non-parametric—between the groups was assessed using the Mann–Whitney *U*-test. The statistical significance of categorical variables was assessed using a Chi-square or Fisher’s exact test. Next, multivariate analysis was performed. A binary logistic regression model was developed to identify the factors that were associated with success after a single protocol run. The model included covariates that showed an association in the univariate analysis of *P *< 0.5 or factors known to contribute to success from previous studies. A stepwise forward method was used to determine the optimal multivariate model. *P* values of < 0.05 were considered to be statistically significant. Unadjusted and adjusted odds ratios (OR) were reported with 95% confidence intervals (CI).

## Results

### Subject identification and baseline characteristics

In total, 91 subjects who had been treated for FRI were identified: 49 for protocol *A* and 42 for protocol *B*. Ten subjects per protocol were excluded because of major deviation. The remaining 71 subjects were eligible for analysis. The majority (75%) of subjects were males, with a median age of 44 years. The pelvis and lower extremities were most often affected. The fractures were open in 45% of subjects, and most had been treated by nailing or plating (see Table 1).

**Table 1:** Baseline characteristics and association between multiple patient-related factors and protocol

Variable	Total (*n *= 71)	Protocol		*P* value	Missing
		*A* (*n* = 39)	*B* (*n* = 32)		
Patient					
Age (years)	44 (17–73)	45(18–69)	38 (17–73)	0.278^c^	0
Male gender	53	29	24	0.951^a^	0
Smoking	28	17	11	0.749^a^	6
Comorbidities					
Diabetes mellitus	8	4	4	1.0^b^	0
Psychiatric disorder	18	9	9	0.627^a^	0
Multitrauma	32	20	12	0.246^a^	0
NSAID use during treatment	52	27	25	0.278^a^	1
Oral corticosteroid use	4	1	3	0.310^b^	2
Follow-up (months)	15 (2–111)	11 (3–32)	23 (2–111)	**0.003**^c^	0
Fracture					
Localization				0.092^b^	0
Sternum/costa	1	1	0		
Humerus	2	1	1		
Radius/ulna	6	3	3		
Pelvic ring/acetabulum	12	8	4		
Femur	15	7	8		
Tibia/fibula	19	11	8		
Ankle	8	6	2		
Foot	8	2	6		
Soft tissue injury				0.075^a^	1
Closed and Gustilo I	47	29	18		
Gustilo II and III	23	9	14		
Fracture fixation					
Treatment before definite fixation				0.712^b^	0
Cast	5	4	1		
External fixation	15	7	8		
Time until fixation				**0.001**^a^	3
0–6 h	19	3	16		
6–24 h	13	10	3		
1–7 days	13	10	3		
> 1 week	23	13	10		
Type of osteosynthesis				0.514^b^	0
Zuggurtung/K-wire	3	1	2		
Screws	10	4	6		
Nail	18	9	9		
Plate	40	25	15		
Closure					0
Primary closure	56	30	26	0.952^a^	
Direct free flap transplantation	4	2	2	1.0^b^	
Secondary closure, including vacuum therapy	11	4	7	0.204^b^	
Time until secondary closure, days	129 (3–399)	79 (3–399)	195 (22–276)	0.592^c^	

Bold *P* values are < 0.05 and considered significant

Data are presented as the number of cases or as median with the range in parenthesis

^a^Pearson Chi-square

^b^Fisher’s exact

^c^Mann–Whitney *U*-test

### Treatment characteristics

Treatment for infection started within a week of the first signs of infection in most of the subjects. The median number of debridements was 2 (1–22) and did not differ between both protocols. Local antibiotics were used in 54% and did not differ between the two protocols. After debridement, 54% of the wounds were primarily closed. In protocol *B*, significantly more direct skin or flap transplantations were executed (*P *= 0.009). Furthermore, the median duration of both intravenous and oral antibiotic administration differed significantly between the protocols (*P *= 0.000). However, the median total duration of the antibiotic treatment was 80 days (7–202) and did not differ significantly (*P *= 0.235; see Table 2).

**Table 2:** Baseline characteristics and association between variables of treatment of the infection and protocol

Treatment variable	Total (*n *= 71)	Protocol		*P* value	Missing
		*A* (*n* = 39)	*B* (*n* = 32)		
Duration symptoms of infection before treatment				0.146^b^	0
< 1 day	13	8	5		
1–7 days	47	28	19		
1–3 weeks	11	3	8		
Number of debridements performed	2 (1–22)	2 (1–14)	3 (1–22)	0.209^c^	
Local Antibiotics with debridement^d^	38	19	19	0.370^a^	0
Closure after debridement					0
Primary closure	38	21	17	0.952^a^	
Secondary closure	24	17	7	0.952^a^	
Transplantation (flap/skin)	9	1	8	**0.009**^b^	
Vacuum therapy^e^	29^1^	12	17	0.057^a^	
Time to secondary closure after debridement, days^f^	59 (6–540)	75 (9–540)	26 (6–214)	0.101^c^	
Antibiotic therapy					0
Duration i.v. antibiotic therapy	17 (7–116)	10 (7–116)	28 (7–110)	**0.000**^c^	
Duration oral antibiotic therapy	57 (0–119)	70 (0–112)	28 (0–119)	**0.000**^c^	
Duration total antibiotic therapy	80 (7–202)	81 (7–126)	64 (14–202)	0.235^c^	
Duration of rifampicin therapy	42 (0–116)	80 (7–116)	0 (0–93)	**0.000**^c^	

Bold *P* values are < 0.05 and considered significant

Data are presented as the number of cases or as median with the range in parenthesis

^a^ Pearson Chi-square

^b^Fisher’s exact

^c^Mann–Whitney *U*-test

^d^Local antibiotics consisted of either gentamicin-loaded beads, cement, or bioresorbable films in both protocols and were administered at assessment of the treating physician

^e^Overlap with secondary closure and skin or flap transplantation

^f^Outliers in wound closure in both groups were due to small skin defect with diminished wound-healing tendencies. Vacuum or antibiotic therapy was not indicated for the total duration needed for closure

### Success

Primary success was achieved in 72% of the subjects in protocol *A* and in 47% in protocol *B* (*P *= 0.033). The overall success rate was 90% and 78%, respectively, for each protocol. The time between trauma and success did not differ statistically between protocols, with a median duration of 7 months (range 2–37) in protocol *A* and a median of 16 months (range 1–35, *P *= 0.097) in protocol *B*. In contrast, the time between infection and success differed significantly, with 5 months (range 2–25) for protocol *A* and 14 months (range 1–33) for protocol *B* (*P *= 0.007; see Table 3).

**Table 3:** Association between treatment outcome and protocol

	Baseline	Protocol		*P* value
	(*n *= 71)	*A* (*n* = 39)	*B* (*n* = 32)	
Primary success	43	28	15	**0.033**^a^
Overall success	60	35	25	0.204^b^
Time from trauma until success (months)	10 (1–37)	7 (2–37)	16 (1–35)	0.097^c^
Time from infection until success (months)	7 (1–33)	5 (2–25)	14 (1–33)	**0.007**^c^

Bold *P* values are < 0.05 and considered significant

Data are presented as the number or as median with the range in parenthesis

^a^Pearson Chi-square

^b^Fisher’s exact

^c^Mann–Whitney *U*-test

Several variables were selected for logistic regression, and were key elements from both treatment protocols, or factors associated with a decreased chance of success as determined in the literature. The patient-related elements of gender, age, Gustilo grade and polytrauma were included, as were the key elements of protocol, primary closure, rifampicin treatment and duration of antibiotic treatment. A step-by-step logistic regression was performed and resulted in a simple model with the different protocol elements adjusted for both age and Gustilo grade. The other factors did not contribute significantly. Because of the limited number of patients with failure of success included in this study, in order to retain optimal adjustment, no further covariates were added. The crude and adjusted ORs and 95% CI for success for each protocol element are presented in Table 4. The separate elements of the protocols did not yield statistical significance for the prediction of success after adjustment treatment with rifampicin was significantly associated with success (OR 3.28, CI 1.03–10.5, *P *= 0.045). Treatment according to protocol *A* predicted a higher chance of success than with protocol *B* (OR 2.89, CI 1.08–7.72, *P *= 0.035) and remained significant after adjustment for age and Gustilo grade (OR 3.45, CI 1.13–10.52, *P *= 0.030). After adjustment for age and Gustilo classification, the administration of rifampicin contributed to higher success rates (OR 3.28, CI 1.03–10.5, *P *= 0.045). Primary closure and the duration of antibiotic treatment did not contribute to different success rates (see Table 4).

**Table 4:** Crude and adjusted odds ratios of key elements of the treatment protocols and success

Regimen variable	Crude odds ratio			Adjusted odds ratio^a^		
	OR	95% CI	*P* value	OR	95% CI	*P* value
Total protocol *A*	2.89	1.08–7.72	**0.035**	3.45	1.13–10.52	**0.030**
Primary closure	2.61	0.98–6.94	0.055	2.09	0.73–5.98	0.171
Rifampicin treatment	2.52	0.92–6.93	0.073	3.28	1.03–10.5	**0.045**
Duration of total antibiotic treatment	0.99	0.97–1.0	0.136	0.99	0.97–1.01	0.208

Bold *P* values are < 0.05 and considered significant

^a^Adjusted odds ratios were all adjusted for age and Gustilo classification

### Microbial culture and resistance

Cultures were obtained from 70 subjects, of which five showed no growth. Of the positive cultures, 43 showed *Staphylococcus aureus*, of which 29 were monomicrobial and 13 were found in polymicrobial cultures. Nine cases exhibited monomicrobial cultures with other bacteria (Table 5). Follow-up cultures were obtained in 56 subjects (79%), with 44 positive cultures with comparable distribution as the initial cultures. There was no significant difference in the bacteria cultured (e.g., *S. aureus,* CoNS, polymicrobial) in the two protocols (*P *= 0.213), nor did the percentage of initial or follow-up resistance differ (*P *= 0.969; Tables 6, 7). In protocol *A*, vancomycin was effective in 85% of the cases (29/34 positive cultures) for all or part of the cultured bacteria. Protocol *A* showed more rifampicin resistance and protocol *B* more amoxicillin/clavulanic acid resistance, corresponding to the antibiotics prescribed regularly in the respective protocols.

**Table 5:** Microbiological characteristics and hospital

Microbiological variable	All *n *= 71)	Protocol		*P* value	Missing	All (*n *= 71)	Protocol		*P* value	Missing
		*A* (*n* = 39)	*B* (*n* = 32)				*A* (*n* = 39)	*B* (*n* = 32)		
First culture					1					15
Negative culture	5	5	0	0.058^b^		14	8	6	0.212^a^	
*Staphylococcus aureus*	29	13	16	0.182^a^		10	3	7	0.489^b^	
*Staphylococcus epidermidis*(CoNS)	1	1	0	1.0^b^		0	0	0	1.0^b^	
Polymicrobial infections	27	16	11	0.508^a^		26	10	16	0.698^a^	
Other single microbial infections^c^	8	3	5	0.455^b^		8	3	5	1.0^b^	

Data are presented as the number of cases

^a^Pearson Chi-square

^b^Fisher’s exact

^c^*Enterococcus faecalis, Enterobacter gergoviae, Enterobacter cloacae complex, Citrobacter koseri, Klebsiella oxytoca,* Gram negative bacteria, *Candida albicans, Pseudomonas aeruginosa, Streptococcus constellatus, Escherichia coli, Clostridium* spp

**Table 6:** Microbiological resistance per protocol

Microbiological variable	No. samples for analysis (*n*)^c^	Protocol^c^		*P* value	Missing
		*A*	*B*		
Initial resistance	69	5/38	1/31	0.213^b^	0
Initial rifampicin resistance	46	1/28	0/8	1.0^b^	10
Follow-up resistance	42	2/16	5/25	0.685^b^	1
Follow-up rifampicin resistance	24	2/11	0/5	1.0^b^	8
Overall resistance	69	6/38	5/31	0.969^a^	0
Overall rifampicin resistance	47	3/31	0/8	1.0^b^	9

^a^Pearson Chi-square

^b^Fisher’s exact

^c^The numbers of samples (denominator) indicated in the second column are all the positive cultures on which susceptibility testing was applicable. In the missing cases susceptibility testing was not performed on the prescribed antibiotics. Positive samples per hospital were presented as a fraction of total tested for susceptibility

**Table 7:** Microbiological resistance per bacterium

Micro-organism	Antibiotic	Protocol
Initial resistance		
CoNS	Ciprofloxacin	*A*
*Staphylococcus epidermidis*	Ciprofloxacin, rifampicin*	*A*
*Enterobacter gergoviae*	Amoxicillin/clavulanic acid	*A*
*Stenotrophomonas maltophilia*	Ciprofloxacin	*A*
*Staphylococcus epidermidis*	Tetracyclin	*A*
*Staphylococcus aureus*	Cefazolin**	*B*
Follow-up resistance		
CoNS	Rifampicin	*A*
*Staphylococcus epidermidis*	Flucloxacillin, rifampicin*	*A*
*Enterobacter cloacae*^a^	Amoxicillin/clavulanic acid	*B*
*Escherichia coli*	Amoxicillin/clavulanic acid	*B*
*Staphylococcus aureus*	Clindamycin	*B*
*Staphylococcus aureus*	Cefazolin**	*B*
*Escherichia coli*	Amoxicillin/clavulanic acid, ciprofloxacin	*B*

All bacteria cultured which showed resistance for administered antibiotics were included in this table

Antibiotics indicated with an astrisk (* respectively **) were overlapping in resistance patterns in initial and follow-up resistance

^a^Although most wild-type strains of *Enterobacter cloacae* are amoxicillin-clavulanic acid resistant, in this case a susceptible strain was cultured before resistance emerged

## Discussion

The aim of this study was to compare the effect of two standardised treatment regimens for (sub)acute FRI that were similar in their philosophy for aggressive surgical debridement and implant retention, but differed in their use of antibiotics. The immediate administration of an antibiotic directed against the most common pathogens in combination with rifampicin seemed to contribute to a higher rate of success. In addition, an early change to oral antibiotics did not lead to worse outcomes in terms of success or antibiotic resistance in protocol *A*. However, as these elements are totally integrated in the protocols, their individual effects cannot be distinguished based on this data. Despite better logistics and earlier soft tissue coverage in protocol *B*, the primary success rate was significantly higher in protocol *A* (*A* = 72% and *B* = 47%), which could be attributed to the treatment protocol as a whole and the liberal use of rifampicin (adj-OR 3.45, CI 1.13–10.52). As the antibiotic strategy was the most prominent difference between the two protocols in the logistic regression analysis, this supports the argument for the early use of empirical antibiotics in the treatment of acute FRI.

As evidenced in our study, the majority of bone and joint infections are caused by gram-positive bacteria, with *S. aureus* as the most common [3, 9]. However, in the case of the exogenous infection of a fracture site, for example in open fractures or perioperative contamination, CoNS are more common. This is thought to be due to contamination by skin flora and the increased pathogenicity of low-virulent microorganisms caused by the presence of the implant material [13, 14] and/or the decreased microperfusion of injured tissue. In our study, a relatively high number of patients with polymicrobial cultures were observed [3]. This could be due not only to the relatively high number of complicated fractures, but also to the contamination of superficial wound swabs. Therefore, for future microbiological identification, only deep tissue samples are recommended [15]. Nonetheless, we studied two protocols that represented clinical practice, and as the scope of this study was the effect of the treatment rather than the microbiological identification, we chose not to exclude swab samples from this study. Biofilm-forming bacteria are a common cause of FRI and require special attention in terms of a treatment approach [8]. For example, planktonic *S. aureus* can begin to form a biofilm within 24 h. In a biofilm, bacteria form an extracellular matrix—which hinders antibiotic penetration—and enter a stationary growth phase, resulting in decreased antibiotic susceptibility [16]. We attempted to exclude infections with mature biofilms by limiting the study to acute infections (< 3 weeks). However, it is possible that, as more delayed infections were included in protocol *B*, dormant infections led to the unnoticed maturation of biofilm before symptoms occurred. If this is the case, it could have contributed to the lower primary success rates in protocol *B*. It has been recognised that the successful treatment of mature biofilm infections consists not only of antimicrobial, but also of a surgical approach to reduce bacterial load, for which implant replacement is sometimes necessary [13]. Biofilms in their early stages (< 10 days) are significantly less resilient to antibiotic eradication than older biofilms [17]. The appropriate antibiotic treatment has been demonstrated to be able to eradicate young biofilms entirely without the need for mechanical reduction of the biofilm [18–20]. The use of local antibiotics permits immediate availability and may thereby contribute to the eradication or destabilisation of young biofilms [21]. Thus, with a vigorous antibiotic approach directly following initial debridement, one might maintain control of the local situation. As the majority of implant-related infections are caused by Staphylococci, empirical therapy must possess antimicrobial properties for at least gram-positive bacteria.

Rifampicin is an example of an antibiotic with favourable pharmacokinetic properties for biofilm infections because of its high bioavailability and capacity to affect bacteria in the stationary phase [22]. A disadvantage of rifampicin use is the quick emergence of (cross-)resistance, mainly when it is administered as monotherapy or in cases with high bacterial load [23, 24]. In this study, we found one new case of rifampicin resistance. This low rate of emergence of new resistance is in line with the literature, as rifampicin was not administered as monotherapy in either of the protocols, and thorough surgical debridement minimalised bacterial load prior to administration. Although we believe that the addition of rifampicin to the antibiotic therapy makes a valuable contribution, perhaps stricter indications should be maintained.

Amoxicillin/clavulanic acid resistance was found most frequently in the patients who had been treated according to protocol *B*, in line with the most frequent administration of this drug in the corresponding protocol. Resistance emerged twice in *Escherichia coli*. With high rates of the prescription of antibiotics in primary care facilities, the resistance of bacteria is a growing problem [25]. Although the Netherlands has relatively low bacterial resistance, broad-spectrum penicillin, like amoxicillin/clavulanic acid, is prescribed most often and correlates with higher resistance rates [25]. Studies have demonstrated that amoxicillin/clavulanic acid resistance in *E. coli* has risen to 14% in the past decades [26]. In addition, resistance has been observed in commensal skin flora, such as wild-type *S. aureus* and *epidermidis*, which have 5% and 14% resistance against rifampicin, respectively [27]. Taking this into account, we are unable to claim with certainty whether our findings demonstrate the emergence of new resistance or merely reflect the selection of already present resistant mutants. Furthermore, in 40% of the cases, there was no information on follow-up cultures (20% missing, 20% negative cultures), and thus definitive statements on the emergence of resistance could not be made.

Both protocols were intended to achieve early surgical debridement and wound closure at the initial fracture fixation. Several studies have described the benefits of primary closure in fracture treatment. Both Scharfenberger et al. [28] and Jenkinson et al. [29] showed that there are lower infection and non-union rates in primary closures after open fractures than after delayed closures. In our study, we did not find any advantages of primary closure. The high numbers of primary closures, as intended in both protocols, may have hindered statistical significance in this cohort.

When primary closure was feasible, a drain was left in protocol *B*. This has been considered in the literature to have both advantages (fluid and hematoma drainage) and disadvantages (a conduit to the external environment) [30]. Parker et al. [31] showed in a meta-analysis that there is no benefit to closed suction drainage after orthopaedic surgery in terms of infection, hematoma, dehiscence or re-operations, while they demonstrated that there is a higher requirement for blood transfusion when drains are applied [31]. When primary closure could not be achieved, direct flap coverage was preferred in protocol *B* and vacuum dressing in protocol *A*, with secondary wound healing or delayed flap coverage in extensive soft tissue damage. Direct flap coverage of severe soft tissue injuries that accompany fractures can be executed safely, whereas delayed (> 72 h) free flap transplantation may be associated with more wound complications [32, 33]. Conversely, others have advocated that staged soft tissue management minimises swelling and thereby reduces post-reconstructive wound complications [34]. Vacuum dressing may contribute to reduction in the need for flap transfer, size and reoperation, but uncertainty exists as to whether the beneficial effects extend beyond the first week after initiation [35]. In this cohort, we could not confirm nor counter these findings.

There are some limitations to this study. Since this was a retrospective cohort, although patients’ baseline characteristics were similar, fracture treatment characteristics were not standardised. This resulted in differences in, e.g., free flap transplantations (timing/frequency) and time until definitive fixation that could not be analysed separately because of their small numbers and integration in the rest of the protocol. This also applies to the analysis of the independence of separate elements of antibiotic treatment. The major differences between the two protocols were the timing of the start and the choice of antibiotics. Both, protocol *A,* as a whole, and the rifampicin treatment, as a separate element, were predicted to have higher success rates. However, due to the limited number of failures in this study and the incorporation of the rifampicin in protocol *A*, the aforementioned elements could not be analysed separately. Second, not all patients had routine follow-up cultures. Although all clinically relevant, redebridement or protracted infection follow-up tissue samples were obtained, additional samples from all treated patients could contribute to further insight into resistance patterns. Last, the follow-up time was significantly longer in protocol *B*. Although this is mostly explained by the prolonged inclusion period and longer time-until-success, it may lead to higher relapse rates and thereby result in lower success rates for protocol *B*. In addition to methodological limitations, since the execution of this study, a consensus on the definition of FRI has been reached and published [15]. Although this study did not adhere to the strict criteria formulated in the consensus paper, it reflects clinical practice at the time in question, and its findings are still valuable.

In conclusion, overall success rates were high in this setting, where source control and soft tissue coverage were the basis of both treatment protocols. Direct empirical combination antibiotic therapy that consisted of vancomycin and rifampicin yielded higher success rates than delayed narrow-spectrum antibiotics. It is also evident that the former treatment can be used safely, as both protocols had low rates of emerging bacterial resistance. Early combination antibiotics that are directed at the most common pathogens should be considered as the standard of care in acute FRIs.
